# Polymorphisms in GSTM1, GSTT1 and CYP1A1 and risk of pancreatic adenocarcinoma

**DOI:** 10.1054/bjoc.2000.1221

**Published:** 2000-04-27

**Authors:** G Liu, P Ghadirian, D Vesprini, N Hamel, A-J Paradis, G Lal, S Gallinger, S A Narod, W D Foulkes

**Affiliations:** Centre for Research in Women's Health, Women's College Hospital, University of Toronto, Toronto, M5G 1N8, Canada; Centre de Recherche, Pavillon Hotel Dieu, CHUM, Montreal, Quebec, H2W 1T8, Canada; Department of Medicine, Montreal General Hospital Research Institute, McGill University, Montreal, Quebec, H3G 1A4, Canada; Samuel Lunenfeld Research Institute, Mount Sinai Hospital, University of Toronto, 600 University Avenue, Toronto, M5G 1X5, Canada

**Keywords:** pancreatic cancer, genetic susceptibility, cytochrome P450, glutathione S-transferase

## Abstract

A prospective study of 149 unselected incident cases of pancreatic adenocarcinoma and 146 ethnically-matched controls found no associations between *GSTM1* (adjusted odds ratio (AOR) 1.14), *GSTT1* (AOR: 1.19) and *CYP1A1* (AOR: 1.08) polymorphisms and pancreatic cancer susceptibility. Smoking and drinking status did not affect results. These polymorphisms do not appear to be important gene modifiers in pancreatic cancer. © 2000 Cancer Research Campaign


*British Journal of Cancer* (2000) **82**(10), 1646–1649
© 2000 Cancer Research Campaign
DOI: 10.1054/ bjoc.2000.1221, available online at http://www.idealibrary.com on IDEAL®

### Polymorphisms in GSTM1, GSTT1 and CYP1A1 and risk of pancreatic adenocarcinoma

**G Liu[1], P Ghadirian[2], D Vesprini[1], N Hamel[3], A-J Paradis[2], G Lal[4], S Gallinger[4], SA Narod[1] and WD Foulkes[3]**

[1]Centre for Research in Women's Health, Women's College Hospital, University of Toronto, Toronto, Canada, M5G 1N8; [2]Centre de Recherche, Pavillon Hotel Dieu, CHUM, Montreal, Quebec, Canada, H2W 1T8; [3]Montreal General Hospital Research Institute, Department of Medicine, McGill University, Montreal Quebec, Canada, H3G 1A4; [4]Samuel Lunenfeld Research Institute, Mount Sinai Hospital, 600 University Avenue, University of Toronto, Toronto, Canada, M5G 1X5

**Summary** A prospective study of 149 unselected incident cases of pancreatic adenocarcinoma and 146 ethnically-matched controls found no associations between *GSTM1* (adjusted odds ratio (AOR) 1.14), *GSTT1* (AOR: 1.19) and *CYP1A1* (AOR: 1.08) polymorphisms and pancreatic cancer susceptibility. Smoking and drinking status did not affect results. These polymorphisms do not appear to be important gene modifiers in pancreatic cancer. © 2000 Cancer Research Campaign

**Keywords:** pancreatic cancer; genetic susceptibility; cytochrome P450; glutathione S-transferase

Phase I P450 cytochromes and phase II glutathione-S-transferases are supergene families involved with carcinogen metabolism. Polymorphic variants at the *GSTM1*, *GSTT1* and *CYP1A1* loci have been implicated in cancer risk for a number of cancers including lung, bladder, gastrointestinal tract, skin, endometrium and breast cancer (Xu et al, 1996; Rebbeck, 1997). As smoking is a risk factor for pancreatic cancer, and highly penetrant genes have a minor role in pancreatic aetiology (Flanders and Foulkes, 1996), it is possible that a substantial population attributable risk could be contributed by these genes. The present study was therefore undertaken to examine *GSTM1*, *GSTT1* and *CYP1A1* polymorphisms as potential molecular markers of pancreatic cancer susceptibility.

### MATERIALS AND METHODS

### Study population

Patients with newly diagnosed pancreatic cancer were enrolled from the inpatient units, and outpatient cancer clinics of nine tertiary care hospitals in Toronto and Montreal from July 1996 to October 1998. Eligible adult patients received a histologically confirmed diagnosis of pancreatic adenocarcinoma. A total of 161 cases were enrolled of 204 eligible cases (79% participation rate), with patient refusal and terminal care being major reasons for non-participation. Nine enrolled cases had significant missing genotype or interview information, two withdrew participation, and in one case had already another sibling enrolled. So, 149 cases were analysed.

For each patient, a spouse or unrelated family member (when there was no spouse) from the same generation as the case (e.g. brother-in-law) was selected as a control ($n$ = 103). For those with no family member control available ($n$ = 43), an age-, gender- and ethnically-matched population control was chosen. The population controls were identified from a group of individuals recruited by telephone from random-digit dialling techniques. Three cases were multi-ethnic and no appropriate controls were found.

For each case and control, a questionnaire was administered either in person or by telephone. Subjects were asked about their age, smoking history, drinking history and past medical history. Self-reported ethnicity was utilized through assessment of grandparents' heritage and place of birth. Interviews were standardized using scripted texts and standardized prompts. All subjects provided a blood specimen. The protocol was approved by the institutional review board of each hospital or university, and informed consent was obtained from all participants.

### Laboratory analysis

Polymerase chain reaction (PCR) amplifications were performed from genomic DNA extracted from blood for all patients and controls using standard methods. Genotyping of *GSTM1* and *GSTT1* were performed by published methods (Zhong et al, 1993; Pemble et al, 1994). In both cases, presence of an internal control product concurrent with the absence of a *GSTM1*- or *GSTT1*-specific product was indicative of homozygosity for the null allele. The *CYP1A1* genotype was determined by PCR using allele-specific primers of the isoleucine-valine polymorphism in residue 462 in exon 7 according to a modification of the method previously described (Rebbeck et al, 1994). Because the Msp polymorphism of *CYP1A1* is tightly linked with the Ile-Val substitution, and is rare (approximately 1%) in non-Japanese cohorts, only the Ile-Val substitution was evaluated (Hirvonen, 1995). All PCR assays were done without knowledge of case or control status.

### Statistical analysis

*Methods*

Conditional logistic regression, excluding ethnically unmatched cases, was used to estimate the initial odds ratios. However,

*Received 22 July 1999*
*Revised 22 November 1999*
*Accepted 24 January 2000*

*Correspondence to:* G Liu, Dana 1056, Dana-Farber Cancer Institute, 44 Binney Street, Boston, MA 02115, USA

because conditional logistic regression yielded virtually identical results to unmatched logistic regression, the final analysis performed was an unmatched logistic regression, using the SAS program, and included all cases and controls. The following factors were controlled for in the analysis: age (within 5 years), gender, centre attended (Toronto vs Montreal), ethnicity, smoking and drinking status. Interactions among the different polymorphisms and smoking were performed as secondary analyses.

### Power

At a two-sided α (alpha) of 0.05, with 150 cases and 150 controls, where the expected controls had a prevalence of the null genotype of 50% for *GSTM1*, 20% for *GSTT1* and 20% for the variant *CYP1A1*, we have an 80% power to detect an odds ratio for pancreatic cancer risk between genotypes of ≥ 1.7 (*GSTM1*); ≥ 2.0 (*GSTT1* and *CYP1A1*).

### RESULTS

The study population is described in Table 1. The ratio of males and females in the cases is similar to that observed in the Canadian population with pancreatic cancer. There were no differences in ethnicity mix, gender, or age between cases and controls. There were trends for more smokers ($P = 0.06$) and drinkers ($P = 0.07$) to have pancreatic cancer. Pancreatitis and diabetes were more frequent in the cases, but this effect disappeared when only those conditions which appeared more than 3 years before the time of diagnosis were considered ($P > 0.20$ for both conditions), suggesting that these medical conditions were actually the first manifestations of pancreatic cancer.

Genotype data are provided in Table 2. There were no differences ($P > 0.60$ for all genotypes) between cases and controls in *GSTM1*, *GSTT1* and *CYP1A1* genotype distribution. The prevalence of the control null or variant genotypes is similar to those found in other Western population studies (Hirvonen, 1995; Rebbeck, 1997). No differences were found when the data were stratified by ethnicity.

The overall adjusted odds ratio for pancreatic cancer with the *GSTM1* null genotype was 1.14 (95% confidence interval (CI) 0.71–1.81), *GSTT1* null genotype 1.19 (95% CI 0.66–2.16), and *CYP1A1* variant, 1.08 (95% CI 0.51–2.14), adjusting for drinking, smoking and ethnicity. The unadjusted odds ratios (*GSTM1* null 1.13; *GSTT1* null 1.19; *CYP1A1* variant 1.08) were similar to the adjusted odds ratios. In the logistic regression analyses, smoking and drinking status, ethnicity, and genotype status were not found to influence the development of pancreatic cancer. The *P*-values for all models examined were greater than 0.20. Subset analyses did not show any interactions amongst the different polymorphisms and the development of pancreatic cancer.

**Table 1** Characteristics of the study population

| | Cases | | Controls | | |
|---|---|---|---|---|---|
| | *n* | (%) | *n* | (%) | *P*-value[a] |
| Median age, years (range) | 66 (24–83) | | 64 (29–77) | | n.a. |
| Gender | | | | | |
| Male | 79 | (53) | 76 | (52) | n.a. |
| Female | 70 | (47) | 70 | (48) | |
| Ethnicity[b] | | | | | |
| French/French Canadian | 63 | (42) | 63 | (43) | n.a. |
| British/Irish | 39 | (26) | 39 | (27) | |
| Other European | 23 | (15) | 23 | (16) | |
| Ashkenazi Jewish | 13 | (8) | 13 | (8) | |
| Asian/Arab | 8 | (5) | 8 | (5) | |
| Other/multi-ethnic | 3 | (2) | 0 | (0) | |
| Smoking status[c] | | | | | |
| Never | 84 | (56) | 96 | (66) | 0.06 |
| Light | 33 | (22) | 30 | (26) | |
| Heavy | 32 | (21) | 20 | (15) | |
| Drinking status[d] | | | | | |
| Never | 67 | (45) | 85 | (58) | 0.07 |
| Light | 45 | (30) | 32 | (22) | |
| Heavy | 37 | (25) | 29 | (20) | |
| Pancreatitis | | | | | |
| Ever | 9 | (6) | 0 | (0) | 0.004 |
| > 3 years before diagnosis | 3 | (2) | 0 | (0) | 0.25 |
| Diabetes mellitus | | | | | |
| Ever | 13 | (9) | 3 | (2) | 0.02 |
| > 3 years before diagnosis | 5 | (3) | 3 | (2) | 0.73 |

[a]n.a. = non-applicable; $\chi^2$ trend test for smoking and drinking; $\chi^2$ tests or Fisher's exact tests for all other categories. [b]Ethnicity was defined as having at least three grandparents in the same ethnicity category. All others were classified as multi-ethnic. [c]Non-smokers had fewer than 100 cigarettes in their lifetime; light smokers had 30 lifetime pack-years (product of number of packs per day × number of years smoking) or fewer; and heavy smokers, more than 30 pack-years. Twenty-five cigarettes constituted one pack. [d]Light drinkers have 50 or fewer drink-years. One drink was equivalent to one bottle of beer, one glass of wine, or one shot of hard liquor.

*© 2000 Cancer Research Campaign*

**Table 2** Genotype characteristics of study population

| | Cases | | Controls | |
|---|---|---|---|---|
| | **Null/variant** *n* **(%)** | **Present/standard** *n* **(%)** | **Null/variant** *n* **(%)** | **Present/standard** *n* **(%)** |
| GST-M1 | 81 (54) | 68 (45) | 75 (51) | 71 (49) |
| *subgroups*: | | | | |
| French/French Canadian | 36 | 27 | 37 | 26 |
| British/Irish | 21 | 18 | 17 | 22 |
| Other European | 13 | 10 | 9 | 14 |
| Ashkenazi Jewish | 6 | 7 | 7 | 6 |
| Asian/Arab | 3 | 5 | 5 | 3 |
| Other/multi-ethnic | 2 | 1 | 0 | 0 |
| GST-T1 | 30 (20) | 119 (80) | 26 (18) | 119* (82) |
| *subgroups*: | | | | |
| French/French Canadian | 6 | 57 | 11 | 32 |
| British/Irish | 9 | 30 | 7 | 32 |
| Other European | 8 | 15 | 5 | 18 |
| Ashkenazi Jewish | 2 | 11 | 2 | 10 |
| Asian/Arab | 4 | 4 | 1 | 7 |
| Other/multi-ethnic | 1 | 2 | 0 | 0 |
| CYP1A1 | 20 (13) | 129 (87) | 19 (13) | 127 (87) |
| *subgroups*: | | | | |
| French/French Canadian | 7 | 56 | 9 | 54 |
| British/Irish | 4 | 35 | 4 | 35 |
| Other European | 5 | 18 | 4 | 19 |
| Ashkenazi Jewish | 0 | 13 | 0 | 13 |
| Asian/Arab | 3 | 5 | 2 | 6 |
| Other/multi-ethnic | 1 | 2 | 0 | 0 |

[a]One control did not have GST-T1 genotype data.

### DISCUSSION

Although pancreatic cancer is an important cause of cancer death, genetic factors involved with the aetiology of the disease have not been extensively studied. Previous smaller studies found no associations with *CYP1A1* polymorphisms or *GSTM1* null genotypes (Lee et al, 1997; Bartsch et al, 1998). Our study confirmed a lack of association between pancreatic adenocarcinoma and *GSTT1*, *GSTM1* null-genotypes, or the *CYP1A1* variant. Further, neither smoking status nor alcohol use influenced our results. The strength of the current study is the use of ethnically matched controls. Ethnicity has been shown to greatly affect genotype status (Rebbeck, 1997). The mix of ethnicities in this study allowed for a subset analysis, which showed non-significant risk differences in Caucasian, Jewish or non-Caucasian patients.

The use of spousal controls aimed to decrease the environmental and ethnic differences between cases and controls (Foulkes et al, 1996), and we were successful in obtaining more than half of our controls as spouses. It is this overmatching which possibly led to a non-significant trend for smokers to develop pancreatic cancer in this population ($P$ = 0.06, Table 2).

This study had several limitations. Small and modest differences in risk (relative risks less than 1.7–2.0) would have been missed. The study did not evaluate *GSTM1* subtypes A and B, although there has never been a clear functional difference between these subtypes (Rebbeck, 1997). In vitro studies suggest that the Ile-Val *CYP1A1* polymorphism has no functional consequences (Zhang et al, 1996; Persson et al, 1997) and the functional significance of the *MspI* allele is still unknown. This study did not evaluate the interaction of genotypes and dietary factors. Different dietary factors have been associated with pancreatic cancer, but none consistently (Howe and Burtch, 1996). The potential interactions between several dietary factors and genotypes would be enormous, requiring thousands of pancreatic cancer patients.

In conclusion, we found that *GSTM1*, *GSTT1* homozygous null genotypes and the *CYP1A1* (Ile-Val) genotype are not over-represented in pancreatic cancer patients, and interactions between tobacco and alcohol and polymorphic variation are not observed. There are a number of reasons for a lack of association between these polymorphisms, smoking, and the development of pancreatic cancer. First, *GSTM1*, *GSTT1* and *CYP1A1* may not be among the enzymes involved in the metabolism of the carcinogens responsible for carcinogenesis. Repair genes, such as $O^6$-methyguanine-DNA methyltransferase, might be the primary genetic modifiers of pancreatic cancer risk. Secondly, these polymorphisms are themselves inadequate to modify a person’s risk, and require other genetic or environmental modifiers not yet identified. Future studies will need to address these areas of research.

### ACKNOWLEDGEMENTS

We thank Beverly Schmocker, Margot Mitchell, and Jean-Sebastien Brunet for their invaluable help. We thank the physicians at The Toronto Hospital, Princess Margaret Hospital, Mount Sinai Hospital, Hotel-Dieu de Montreal, Hospital Notre-Dame, Hospital St Luc, Montreal General Hospital, Jewish General Hospital, Royal Victoria Hospital for their support. The research in Montreal was supported by a grant from the Cancer Research Society, Incorporated. The research in Toronto was supported by a grant from the National Cancer Institute of Canada. G Liu was supported by a William S Fenwick Research Fellowship.

*© 2000 Cancer Research Campaign*

### REFERENCES

Bartsch H, Malaville C, Lowenfels AB, Maisonneuve P, Hautefeuille A and Boyle P (1998) Genetic polymorphism of N-acetyltransferases, glutathione S-transferase M1 and NAD(P)H:quinone oxidoreductase in relation to malignant and benign pancreatic disease risk. The International Pancreatic Disease Study Group. *Eur J Cancer Prev* **7**: 215–233

Flanders TY and Foulkes WD (1996) Pancreatic adenocarcinoma: epidemiology and genetics. *J Med Genet* **33**: 889–898

Foulkes WD, Brunet JS, Sieh W, Black MJ, Shenouda G and Narod SA (1996) Familial risks of squamous cell carcinoma of the head and neck: restrospective case–control study. *Br Med J* **313**: 716–721

Hirvonen A (1995) Genetic factors in individual responses to environmental exposures. *J Occup Environ Med* **37**: 37–43

Howe GR and Burch JD (1996) Nutrition and pancreatic cancer. *Cancer Causes Control* **7**: 69–82

Lee HC, Yoon YB, Kim CY (1997) Association between genetic polymorphisms of the cytochrome P450 (1A1, 2D6, 2E1) and the susceptibility to pancreatic cancer. *Korean J Intern Med* **12**: 128–136

Pemble S, Schroeder KR, Spencer SR, Meyer DJ, Hallier E, Bolt HM, Ketterer B and Taylor JB (1994) Human glutathione S-transferase theta (GSTT1): cDNA cloning and the characterization of a genetic polymorphism. *Biochem J* **300**: 271–276

Persson I, Johansson I and Ingelman-Sundberg M (1997) In vitro kinetics of two human CYP1A1 variant enzymes suggested to be associated with interindividual differences in cancer susceptibility. *Biochem Biophys Res Commun* **231**: 227–230

Rebbeck TR (1997) Molecular epidemiology of human glutathione S-transferase genotypes GST-M1 and GST-T1 in cancer susceptibility. *Cancer Epidemiol Biomarkers Prev* **6**: 733–743

Rebbeck TR, Rosvold EA, Duggan, DJ, Zhang J and Buetow KH (1994) Genetics of CYP1A1: coamplification of specific alleles by polymerase chain reaction and association with breast cancer. *Cancer Epidemiol, Biomarkers Prev* **3**: 511–514

Xu X, Kelsey KT, Wiencke JK, Wain JC and Christiani DC (1996) Cytochrome P450 *CYP1A1 Msp1* polymorphisms and lung cancer susceptibility. *Cancer Epidemiol Biomarkers Prev* **5**: 687–692

Zhang ZY, Fasco MJ, Huang L, Guengerich F and Kaminsky LS (1996) Characterization of purified human recombinant cytochrome P450A1-Ile462 and -Val462: assessment of a role for the rate allele in carcinogenesis. *Canc Res* **56**: 3926–3933

Zhong S, Wyllie AH, Barnes D, Wolf CR and Spurr NK (1993) Relationship between the GSTM1 genetic polymorphism and susceptibility to bladder, breast and colon cancer. *Carcinogenesis* **14**: 1821–1824

*© 2000 Cancer Research Campaign*
